# Cost-effectiveness of Prophylactic Zika Virus Vaccine in the Americas

**DOI:** 10.3201/eid2512.181324

**Published:** 2019-12

**Authors:** Affan Shoukat, Thomas Vilches, Seyed M. Moghadas

**Affiliations:** Author affiliations: York University, Toronto, Ontario, Canada (A. Shoukat, S.M. Moghadas); São Paulo State University, Botucatu SP, Brazil (T. Vilches)

**Keywords:** Zika virus, microcephaly, vaccination, agent-based simulations, cost-effectiveness, Americas, viruses

## Abstract

Zika virus remains a major public health concern because of its association with microcephaly and other neurologic disorders in newborns. A prophylactic vaccine has the potential to reduce disease incidence and eliminate birth defects resulting from prenatal Zika virus infection in future outbreaks. We evaluated the cost-effectiveness of a Zika vaccine candidate, assuming a protection efficacy of 60%–90%, for 18 countries in the Americas affected by the 2015–2017 Zika virus outbreaks. Encapsulating the demographics of these countries in an agent-based model, our results show that vaccinating women of reproductive age would be very cost-effective for sufficiently low (<$16) vaccination costs per recipient, depending on the country-specific Zika attack rate. In all countries studied, the median reduction of microcephaly was >75% with vaccination. These findings indicate that targeted vaccination of women of reproductive age is a noteworthy preventive measure for mitigating the effects of Zika virus infection in future outbreaks.

After the 2013–2014 Zika virus outbreak in French Polynesia (*1*,*2*), the disease spread to 69 countries and territories worldwide (*3*). The connection of Zika virus infection to prenatal microcephaly and other brain abnormalities (*4*–*6*) raised a public health emergency of international concern in February 2016 (*7*). Although this concern subsided with declining outbreaks in the Americas, a sizable portion of the population in the tropical world remains at risk for Zika virus infection, especially in countries where the primary transmitting vector (the *Aedes aegypti* mosquito) is abundant (*8*). Furthermore, the economic burden of Zika virus infection is estimated to be substantial, ranging from $7 to $18 billion in short-term costs and $3.2 to $39 billion in long-term costs (*9*), which highlights the need for preventive measures.

The potential for future outbreaks and devastating clinical outcomes with long-term sequelae has directed research efforts to develop an effective Zika virus preventive vaccine (*10*–*13*). Several vaccine candidates have now advanced to clinical trials and have been shown to be safe and well tolerated in generating humoral immune responses (*14*,*15*). For the strategic use of a prophylactic vaccine, a vaccine target product profile (VTPP) has been proposed by the World Health Organization and the United Nations Children’s Fund, prioritizing women of reproductive age (15–49 years), including pregnant women (*16*). To inform decisions on implementing the recommended VTPP, we evaluated the cost-effectiveness of a potential Zika virus vaccine in 18 countries in the Americas where the estimated attack rates (i.e., the proportion of the population infected) during the 2015–2017 outbreaks were >2% (*17*,*18*).

## Methods

### Simulation Model

We adopted a previously established agent-based simulation model for the dynamics of Zika virus infection, incorporating both vector and sexual transmission (*19*,*20*). For infection dynamics, the human population was divided into susceptible, exposed and incubating, infectious (symptomatic and asymptomatic), and recovered categories (Appendix Figure 1). We stratified the mosquito population into susceptible, exposed and incubating, and infectious groups. We parameterized the model with country-specific demographics (age and sex distributions and fertility rates), and calibrated it to attack rates (*17*,*18*) estimated for the 2015–2017 outbreaks (Appendix Tables 1–4, Figures 2–4). These attack rates were considered to be the proportion of the population that was infected (representing the level of herd immunity) at the start of simulations for each country in the evaluation of vaccination scenarios. We compiled parameters specific to Zika virus infection in both human and mosquito populations, along with costs associated with the disease and vaccination (Appendix Tables 5, 6). Further details of the model and its implementation are provided in the Appendix; for reproducibility, the computational model can be accessed at https://github.com/affans/zika.

### Infection Outcomes

We considered microcephaly and Guillain-Barré syndrome (GBS) as outcomes of infection. The risk for microcephaly was highest (5%–14%) for infections occurring during the first trimester of pregnancy (which ends at 97 days) and decreased to 3%–5% for infections occurring during the second and third trimesters (*21*–*23*). We set a probability of 0.798 for survival past the first year of life for infants with microcephaly (*24*). Life expectancy of infants with microcephaly who survived the first year of life was reduced by 50%, from 70 years to 35 years, on average (*25*). The risk for GBS with Zika virus infection in adults was 0.025%–0.06% (*26*).

### Vaccination and Cost-effectiveness

We implemented vaccination scenarios corresponding to the recommended strategies in the VTPP (*16*). The vaccination coverage was set to 60% for women of reproductive age at the onset of simulations. For pregnant women in the same age group, the vaccination coverage was set to 80% initially and continued at 80% throughout the simulations. We also considered a vaccination coverage of 10% for other persons 9–60 years of age. In the absence of efficacy data, we assumed that a single dose of vaccine provides a protection efficacy of 60%–90% against infection, which was sampled for each vaccinated person and implemented as a reduction factor in disease transmission. Infection following vaccination (if it occurred) was assumed to be asymptomatic. Furthermore, we assumed that vaccination has no effect on the risk of microcephaly in pregnant women if infection occurred.

For cost-effectiveness analysis, we considered both short- and long-term medical costs specific to each country (Appendix Table 6) (*9*). Short-term costs included physician visits and diagnostic tests for symptomatic Zika virus infection in pregnant women. For microcephaly in infants and GBS in adults, we considered lifetime direct medical costs related to hospitalization, treatment, and other associated outcomes. We quantified the long-term sequelae of microcephaly by disability-adjusted life-years (DALYs) with disability weight (i.e., severe intellectual disability) extracted from the Global Burden of Disease study (*27*). For given vaccination costs per individual (VCPI), we calculated the incremental cost-effectiveness ratios (ICERs) and averaged them over simulations (Appendix). Both DALYs and direct lifetime costs were based on a 3% discounting rate annually (*9*,*25*). For cost-effectiveness analysis, we considered the World Health Organization standards of using the per capita gross domestic product (GDP) as a threshold of willingness to pay (*28*). The vaccination program was considered very cost-effective for ICER values up to the per capita GDP and cost-effective for ICER values up to 3 times the per capita GDP. We also considered a range of willingness to pay values to inform decisions on vaccine cost-effectiveness in settings in which the per capita GDP threshold may not be applicable. Using a nonparametric bootstrap method, we generated cost-effectiveness acceptability curves for each country and performed cost-effectiveness analysis from a government perspective. All costs are reported in 2015 US dollars.

We ran 2,000 Monte Carlo simulations of Zika virus infection dynamics with a scaled-down population of 10,000 persons for each country. Each simulation was seeded with a single case of Zika virus in the latent stage and run for a time horizon of 1 year with a daily time-step, beginning with a high-temperature season. For each simulation, we recorded the daily incidence of infection and disease outcomes and used them for cost-effectiveness analysis, as well as estimating the percentage reduction of microcephaly attributable to vaccination. DALYs were calculated for the lifetime of each case of microcephaly. Only epidemic curves that had >1 secondary cases by the end of simulations were considered in the cost-effectiveness analysis.

## Results

**Figure 1:**
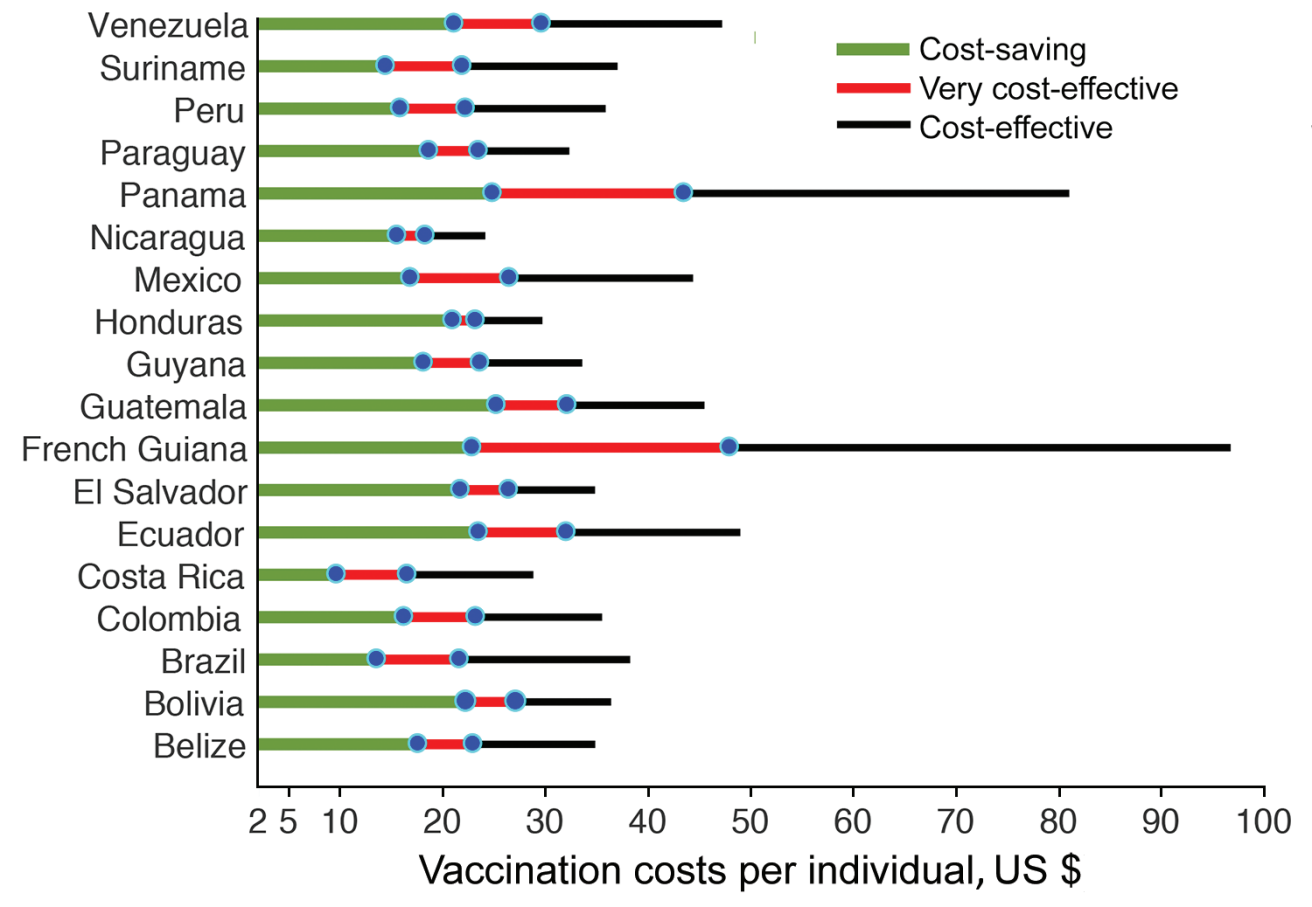
Range of vaccination costs per individual (VCPI; in 2015 US dollars) for the scenarios of whether Zika virus vaccines would be cost-saving (green), very cost-effective (red), and cost-effective (black). All estimates are based on the level of preexisting herd immunity in the population for each country.

We considered a plausible range of $2–$100 for VCPI to account for vaccine dose, wide distribution and administration, and wastage based on the estimates for other flavivirus vaccines (*29*). Our results show that for a sufficiently low VCPI in this range, a single-dose vaccination program is cost-saving for all countries studied (Figure 1, green). The lowest VCPI was found for Costa Rica, where the vaccine was cost-saving with a probability of >90% for VCPI up to $10, derived from the cost-effectiveness acceptability curve (Appendix Figure 5). With the same probability, the highest VCPI under which the vaccine was cost-saving was $25 for Guatemala and Panama. The highest values of VCPI for a cost-saving scenario in other countries were $14–$24.

For positive ICER values, we considered the average per capita GDP of each country in 2015 and 2016 as the threshold for cost-effectiveness (*30*). For this threshold, the vaccine is very cost-effective with a probability >90% at VCPI of <$16 in Costa Rica (mean incremental cost of $7,352/DALY averted; 95% CI $1,280–$9,234/DALY averted) and <$47 in French Guiana (mean incremental cost of $14,475/DALY averted; 95% CI $10,016–$16,653/DALY averted), with other countries having the highest value of VCPI in this range (Figure 1, red). For the threshold of 3 times the per capita GDP, the vaccine is still cost-effective (with a probability of >90%) with VCPI up to $24 (mean incremental cost of $4,829/DALY averted; 95% CI $2,395–$6,068/DALY averted) in Nicaragua and $96 (mean incremental cost of $49,934/DALY averted; 95% CI $36,523–$53,661/DALY averted) in French Guiana, with other countries having the highest value of VCPI in this range (Figure 1, black). We determined the VCPI for scenarios that are cost-saving, very cost-effective, and cost-effective for each country (Table), the corresponding incremental cost per DALY averted with 95% CIs (Table; Appendix Table 7), and the associated cost-effectiveness acceptability curves (Appendix Figure 5).

**Table:** Highest values of VCPI (in 2015 US dollars) for a Zika virus vaccine candidate to be cost-saving, very cost-effective, or cost-effective*

Country	*Herd immunity, %*	*Cost-saving, VCPI*									
			Very cost-effective					Cost-effective			
			GDP	VCPI	ICER	95% CI		3×GDP	VCPI	ICER	95% CI
Belize	21	$18	$4,955	$23	$3,516	$144–$4,575		$14,865	$34	$12,092	$7,379–$15,050
Bolivia	10	$22	$3,097	$27	$1,827	$(872)–$2,669		$9,291	$36	$7,038	$4,249–$9,745
Brazil	18	$14	$8,694	$21	$6,356	$1,596–$7,223		$26,082	$38	$21,725	$14,938–$27,441
Colombia	12	$16	$5,900	$23	$4,184	$1,284–$5,349		$17,700	$35	$14,086	$9,447–$16,736
Costa Rica	2	$10	$11,563	$16	$7,352	$1,280–$9,234		$34,689	$29	$29,061	$15,459–$30,561
Ecuador	8	$24	$6,084	$32	$4,451	$1,343–$5,560		$18,252	$48	$15,581	$10,338–$17,576
El Salvador	16	$22	$3,719	$26	$1,379	$(1,884)–$2,826		$11,157	$34	$8,177	$3,408–$9,785
French Guiana	18	$23	$18,036	$47	$14,475	$10,016–$16,653		$54,108	$96	$49,934	$36,523–$53,661
Guatemala	14	$25	$4,032	$32	$2,544	$148–$3,944		$12,096	$45	$9,786	$6,556–$11,859
Guyana	15	$18	$4,325	$23	$2,270	$(285)–$3,717		$12,975	$33	$10,034	$5,884–$12,262
Honduras	14	$21	$2,358	$23	$892	$(1,711)–$1,705		$7,074	$29	$4,992	$1,623–$6,142
Mexico	5	$17	$8,867	$26	$6,362	$2,564–$7,445		$26,601	$44	$21,652	$14,717–$24,875
Nicaragua	17	$16	$2,109	$18	$595	$(1,465)–$1,231		$6,327	$24	$4,829	$2,395–$6,068
Panama	15	$25	$14,009	$43	$11,001	$7,016–$13,486		$42,027	$82	$37,247	$29,096–$43,898
Paraguay	17	$19	$4,094	$23	$2,348	$(305)–$3,332		$12,282	$32	$9,903	$5,028–$10,670
Peru	4	$16	$6,042	$22	$4,332	$1,087–$4,870		$18,126	$35	$14,028	$9,262 –$16,432
Suriname	22	$14	$7,298	$21	$4,434	$1,505–$6,235		$21,894	$37	$18,705	$12,714–$22,331
Venezuela	19	$21	$7,766	$29	$4,697	$623–$6,590		$23,298	$47	$19,170	$13,160–$23,579

*Mean ICER values with 95% CI correspond to VCPI values under which the vaccination program is at least 90% cost-effective in each country. The per capita GDP and 3 times the per capita GDP were used as thresholds for very cost-effective and cost-effective analyses, respectively. The dollar values in parentheses indicate that the 95% CI extends to negative ICER values, which is considered cost-saving. GDP, gross domestic product; ICER, incremental cost-effectiveness ratio; VCPI, vaccination cost per individual.

**Figure 2:**
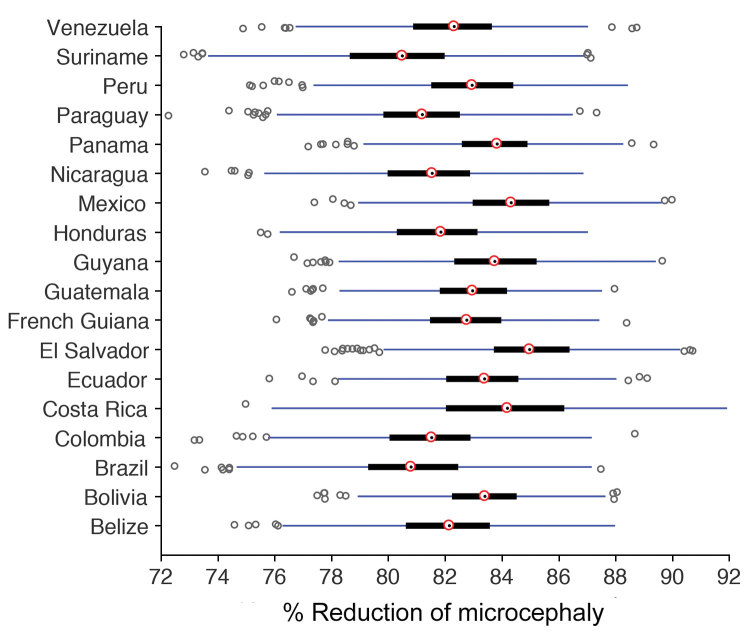
Box plots for the percentage reduction of microcephaly as a result of Zika virus vaccination. Red circles indicate medians; black bars indicate interquartile range (IQR); blue lines indicate extended range, from minimum (25th percentile – 1.5 IQR) to maximum (75th percentile + 1.5 IQR); dark circles indicate outliers.

We also calculated the reduction of fetal microcephaly during pregnancy by comparing the simulation scenarios in the presence and absence of vaccination. We found a marked reduction in cases of microcephaly, within the range of 74%–92%, attributable to vaccination; the median percentage reduction was >80% in all countries (Figure 2). This finding suggests that a Zika virus vaccine with a prophylactic efficacy as low as 60% could substantially reduce the incidence of microcephaly.

Given that the attack rates in future outbreaks may be different from those estimated for the 2015–2017 outbreaks, we further conducted cost-effectiveness analysis for 2 additional scenarios (Appendix Table 8). In the first scenario, we considered an increase of 4% in the estimated attack rate for each country. We found that vaccination was very cost-effective with a probability >90% at VCPI of <$20 in Nicaragua (mean incremental cost of $1,067/DALY averted) and <$50 or less in French Guiana (mean incremental cost of $14,914/DALY averted). The highest VCPI for other countries ranged between these values.

In the second scenario, we decreased the attack rates by 4%, with a lower bound of 1% for each country. The results show that vaccination was very cost-effective, with a VCPI of <$4 in Mexico (mean incremental cost of $3,054/DALY averted) and <$41 in French Guiana (mean incremental cost of $15,037/DALY averted), with other countries having the highest VCPI value in this range (summary of additional results of cost-effectiveness analysis in Appendix Tables 9, 10, and Appendix Figures 6, 7). The median percentage reduction of microcephaly in these scenarios was >75% with vaccination (Appendix Figure 8).

## Discussion

We determined the VCPI within the input range of $2–$100, for which vaccination is cost-saving (when ICER values are negative) and is very cost-effective (when ICER values are positive, below the threshold of the per capita GDP) for 18 countries in the Americas. Although several factors (e.g., the level of preexisting herd immunity, attack rate, costs associated with the management of Zika virus infection and its outcomes, and the willingness to pay) are critical in determining VCPI for cost-effectiveness, our results show that targeted vaccination of women of reproductive age would be cost-effective, and even cost-saving, in all countries studied if VCPI is sufficiently low. Furthermore, vaccination with a protection efficacy of 60%–90% notably reduces the incidence of microcephaly, with a median percentage reduction >75% in simulated scenarios.

Previous work suggests that a prophylactic vaccine with a protection efficacy of 75% reduces the incidence of prenatal infections by >94% if 90% of women of reproductive age are vaccinated (*31*). These estimates are slightly higher than what our model predicts (with a median percentage reduction of 75%–88%) in similar scenarios, which is expected given the deterministic nature of the model used in the previous study (*31*). Nevertheless, the findings indicate that targeted vaccination is a noteworthy preventive measure for mitigating the impact of Zika virus infection in future outbreaks.

Considering direct medical costs associated with short- and long-term Zika virus infection outcomes, our study provides a cost-effectiveness analysis of a Zika virus vaccine candidate from a government perspective. Several recent modeling studies also evaluate cost-effectiveness of a Zika virus vaccine (*20*,*32*). However, these studies have either considered only a few countries in Latin America or relied on homogeneous models. The strength of our study relies on the evaluation of cost-effectiveness for countries affected by Zika virus with estimated attack rates >2% within a single modeling framework. We based our analysis on an individual-level stochastic approach, accounting for parameter uncertainty and heterogeneities in disease transmission. Because of its dynamic nature, the simulation model also considers the accruing herd immunity during the epidemic that results from the indirect protection effects of naturally acquired immunity in the population. 

Our results should be considered within the context of study limitations. First, we note that we based our analysis on estimates of attack rates during the 2015–2017 Zika virus outbreaks in Latin and South America countries (*9*,*17*,*18*), and these attack rates were regarded as the levels of preexisting herd immunity in the simulations. Should these levels change as the result of a decline of herd immunity or accumulation of new susceptible persons at the time of vaccine availability in future outbreaks, the expected changes in the VCPI range for cost-effectiveness require further analysis. Second, although the initial phase of clinical trials indicates high levels of neutralizing antibodies (*14*,*15*), the range of vaccine efficacy has not been ascertained; our estimates rely on the assumption that a single dose of vaccine would provide a protection efficacy of 60%–90%. We assumed that during the epidemic pregnant women are vaccinated (with a coverage of 80%) early in their first trimester, because the highest risk of microcephaly occurs then. However, we understand that because of various factors, including access to healthcare resources and late recognition of pregnancy, vaccination may not occur before any potential Zika virus infection during pregnancy. The risk for microcephaly was not altered if infection occurred following vaccination, but the disease was considered to be asymptomatic. The validation of these assumptions requires efficacy data from clinical trials, which are currently lacking. In our model, the risk of sexual transmission was included only during the infectious period. Although this risk may continue for several days or weeks following recovery (*33*,*34*), our simplifying assumption is justified because of uncertainty in the duration of sexual transmission at the individual level. Despite these limitations, which warrant further investigation as relevant information and data become available, this study provides estimates for Zika virus vaccine cost-effectiveness to inform decision makers for the implementation of the VTPP strategies in an outbreak response scenario.

Appendix Additional information about the model for cost-effectiveness of Zika virus vaccine.
