# Supplementary material for: Cost-effectiveness of Prophylactic Zika Virus Vaccine in the Americas
Source: Emerg Infect Dis. 2019 Dec;25(12):2191–6. doi: 10.3201/eid2512.181324 (PMC6874261; doi:10.3201/eid2512.181324)
Supplement: Appendix — Additional information about the model for cost-effectiveness of Zika virus vaccine. [file 18-1324-Techapp-s1.pdf]

Article DOI: https://doi.org/10.3201/eid2512.181324

Cost-effectiveness of Prophylactic Zika Virus Vaccine in the Americas

Appendix

Model Implementation, Transmission Dynamics, and Disease Outcomes

We adopted a previously established agent-based simulation model of Zika virus infection and vaccination dynamics to evaluate the cost-effectiveness of a Zika virus vaccine in 18 countries in the Americas. The model was parameterized with a scaled-down population of 10,000 humans and 50,000 mosquitoes. Births following pregnancy were considered in the model only implicitly for the effect of microcephaly if Zika virus infection occurred. However, given the short (1-year) simulation timelines, we ignored the individual births and deaths in the populations; therefore, the population size remained constant. The geographic distribution and number of mosquitoes were accounted for in the calibration to country-specific attack rates estimated in previous studies (*1*,*2*). These estimates have accounted for monthly seasonality, with time- and location-dependent variability. Individual age and sex attributes were sampled from their distributions for each country (Appendix Figure 1). Mosquito lifetime was determined through a discretized distribution generated by survival functions, sampled for both high- and low-temperature seasons (*3*).

The model simulates disease spread via 2 main modes of transmission, including vector bites and sexual interactions. Human-to-mosquito transmission (or vice versa) occurred as a result of rejection sampling-based (Bernoulli) trials, where the chance of successful transmission is given by $P_{\text{infection}} = 1 - (1 - \beta)^N$ where N is the number of bites of a single mosquito to an infectious or susceptible individual, and β is the baseline probability calibrated to the Zika attack rate for each country estimated after the 2015–2017 outbreaks (Appendix Table 1). The number of bites for each mosquito was individually sampled from a Poisson distribution with the half-life of the mosquito as the mean of the distribution. The bites over the lifespan of a mosquito were also implemented as a Poisson process with an average of 1 bite every 2 days, and a maximum of

1 bite per day (*3*). Sexual transmission of Zika virus was included in the model for persons >15 years of age and in a monogamous context. The frequency of sexual encounters for partnered persons was sampled from age-dependent distributions (Appendix Tables 2 and 3). For an individual in the age group a_i, the partner was selected from the age group $a_i \pm 5$ years of age.

Upon successful Zika virus transmission, susceptible persons entered an intrinsic incubation period (IIP), sampled for each person from the associated distribution (*6,7*). After the IIP elapsed, a fraction (sampled between 40% to 80%) of infected persons entered asymptomatic infection without developing clinical symptoms (*8,9*). In our previous studies (*3,10*), Zika virus transmission from asymptomatic infection was modeled by a relative transmissibility factor compared with symptomatic infection, which ranged from 0.1 to 0.9. Here we assumed the same transmissibility for both asymptomatic and symptomatic infection, with any transmission reduction in asymptomatic infection accounted for in the calibration process.

Persons who recovered from either asymptomatic or symptomatic infection were assumed to be immune to reinfection for the remainder of the simulation time. A schematic diagram of the model for transmission dynamics, natural history of Zika virus infection, and disease outcomes are provided in Appendix Figure 2. All parameters pertaining to infection dynamics are summarized in Appendix Table 5. Incidence and attack rates for different countries in the absence of vaccination are illustrated in Appendix Figures 3 and 4.

The total number of pregnant women was calculated based on the country-specific fertility rate of population in each simulation (Appendix Table 4). Ignoring fatal complications, the number of pregnant women at any point in time for each simulation was calculated by the following (*14*):

$$\text{Number of pregnant women} = \left(\frac{\text{nWRA}}{1000}\right)(\text{fertility rate } \times\ 0.75 + \text{abortion rate } \times\ 0.167)$$

where nWRA is the number of women of reproductive age, with an abortion rate of 12%. Initial vaccine coverage of women of reproductive age was 60% (at the start of simulations). Initially and during the epidemic simulations, vaccination coverage of pregnant women was set to 80%.

Zika virus-infected persons with symptoms were assumed to incur short- and long-term direct medical costs related to hospitalization, treatment, and long-term sequelae. Costs for different categories are summarized in Appendix Table 6 (*15*). In addition, an average cost of

$150 for Zika diagnostic tests was assumed for pregnant women with symptomatic infection in all countries (*15*). All costs are reported in 2015 US dollars. For cost-effectiveness, we calculated ICER values using the following formula:

$$\text{ICER} = \frac{\text{Cost}_{\text{Vaccination}} - \text{Cost}_{\text{No Vaccination}}}{-(\text{DALY}_{\text{Vaccination}} - \text{DALY}_{\text{No Vaccination}})}$$

Additional Scenarios

Future Zika virus outbreaks may occur with different attack rates from those estimated for the 2015–2017 outbreaks. Therefore, we conducted cost-effectiveness analysis for 2 additional scenarios. In the first scenario, we calibrated the model to an increase of 4% in the estimated attack rate for each country. In the second scenario, the model was calibrated to a 4% decrease in the estimated attack rates, with a lower bound of 1%, for each country. The levels of preexisting herd immunity at the onset of simulations remained the same as those in the Table in the main article.

In the scenario with increased attack rates, the results show that the vaccine is very cost-effective (using per-capita GDP as the threshold) for a VCPI up to $20 in Nicaragua and up to $50 in French Guiana (Appendix Figure 6). The upper VCPI for other countries ranged between these values. Similarly, using 3 times the per capita GDP as the threshold, the vaccine is still cost-effective for a VCPI up to $26 in Nicaragua and up to $98 in French Guiana (Appendix Figure 6). In the scenario with decreased attack rates, the vaccine is (very) cost-effective for a VCPI up to ($4) $9 in Mexico and up to ($41) $84 in French Guiana (Appendix Figure 7), with other countries having an upper VCPI value in this range. Summaries of the cost-effectiveness analysis for both scenarios of higher and lower attack rates are provided in Appendix Tables 9 and 10.

We also calculated the percentage reduction of microcephaly during pregnancy for both scenarios of increased and decreased attack rates. We found that the median percentage reduction in both scenarios was >75% in all countries (Appendix Figure 8).

References

1. Zhang Q, Sun K, Chinazzi M, Pastore Y Piontti A, Dean NE, Rojas DP, et al. Spread of Zika virus in the Americas. Proc Natl Acad Sci U S A. 2017;114:E4334–43. https://doi.org/10.1073/pnas.1620161114

2. Colón-González FJ, Peres CA, Steiner São Bernardo C, Hunter PR, Lake IR. After the epidemic: Zika virus projections for Latin America and the Caribbean. PLoS Negl Trop Dis. 2017;11:e0006007. https://doi.org/10.1371/journal.pntd.0006007

3. Moghadas SM, Shoukat A, Espindola AL, Pereira RS, Abdirizak F, Laskowski M, et al. Asymptomatic transmission and the dynamics of Zika infection. Sci Rep. 2017;7:5829. https://doi.org/10.1038/s41598-017-05013-9

4. Reece M, Herbenick D, Schick V, Sanders SA, Dodge B, Fortenberry JD. Sexual behaviors, relationships, and perceived health among adult men in the United States: results from a national probability sample. J Sex Med. 2010;7(Suppl 5):291–304. https://doi.org/10.1111/j.1743-6109.2010.02009.x

5. Herbenick D, Reece M, Schick V, Sanders SA, Dodge B, Fortenberry JD. Sexual behaviors, relationships, and perceived health status among adult women in the United States: results from a national probability sample. J Sex Med. 2010;7(Suppl 5):277–90. https://doi.org/10.1111/j.1743-6109.2010.02010.x

6. Nishiura H, Halstead SB. Natural history of dengue virus (DENV)-1 and DENV-4 infections: reanalysis of classic studies. J Infect Dis. 2007;195:1007–13. https://doi.org/10.1086/511825

7. Carrington LB, Simmons CP. Human to mosquito transmission of dengue viruses. Front Immunol. 2014;5:290. https://doi.org/10.3389/fimmu.2014.00290

8. Ladhani SN, O’Connor C, Kirkbride H, Brooks T, Morgan D. Outbreak of Zika virus disease in the Americas and the association with microcephaly, congenital malformations and Guillain-Barré syndrome. Arch Dis Child. 2016;101:600–2. https://doi.org/10.1136/archdischild-2016-310590

9. Duffy MR, Chen T-H, Hancock WT, Powers AM, Kool JL, Lanciotti RS, et al. Zika virus outbreak on Yap Island, Federated States of Micronesia. N Engl J Med. 2009;360:2536–43. https://doi.org/10.1056/NEJMoa0805715

10. Shoukat A, Vilches T, Moghadas SM. Cost-effectiveness of a potential Zika vaccine candidate: a case study for Colombia. BMC Med. 2018;16:100. https://doi.org/10.1186/s12916-018-1091-x

11. Gubler DJ, Suharyono W, Tan R, Abidin M, Sie A. Viraemia in patients with naturally acquired dengue infection. Bull World Health Organ. 1981;59:623–30.

12. Chan M, Johansson MA. The incubation periods of dengue viruses. PLoS One. 2012;7:e50972. https://doi.org/10.1371/journal.pone.0050972

13. Salomon JA, Haagsma JA, Davis A, de Noordhout CM, Polinder S, Havelaar AH, et al. Disability weights for the Global Burden of Disease 2013 study. Lancet Glob Health. 2015;3:e712–23. https://doi.org/10.1016/S2214-109X(15)00069-8

14. CDC Division of Reproductive Health. Estimating the number of pregnant women in a geographic area [cited 2018 Jun 1]. https://www.cdc.gov/reproductivehealth/emergency/pdfs/PregnacyEstimatoBrochure508.pdf

15. United Nations Development Programme. A socio-economic impact assessment of the Zika virus in Latin America and the Caribbean: with a focus on Brazil, Colombia and Suriname. 2017 [cited 2017 Jun 1]. https://www.undp.org/content/undp/en/home/librarypage/hiv-aids/a-socio-economic-impact-assessment-of-the-zika-virus-in-latin-am.html

16. Durham DP, Fitzpatrick MC, Ndeffo-Mbah ML, Parpia AS, Michael NL, Galvani AP. Evaluating vaccination stegies for Zika virus in the Americas. Ann Intern Med. 2018;168:621–30. https://doi.org/10.7326/M17-0641

Appendix Table 1. Attack rates estimated for the 2015–2017 Zika virus outbreaks (*1*,*2*), and estimated transmissibility, averaged over 2000 independent simulations for each country.

Country	Attack rate	Estimated transmissibility
Belize	21%	0.2884
Bolivia	10%	0.2761
Brazil	18%	0.2859
Colombia	12%	0.2792
Costa Rica	2%	0.2476
Ecuador	8%	0.2723
El Salvador	16%	0.2839
French Guiana	18%	0.2859
Guatemala	14%	0.2817
Guyana	15%	0.2829
Honduras	14%	0.2817
Mexico	5%	0.2641
Nicaragua	17%	0.2849
Panama	15%	0.2829
Paraguay	17%	0.2849
Peru	4%	0.2602
Suriname	22%	0.2891
Venezuela	19%	0.2868

Appendix Table 2. Age-dependent probability matrix of sexual encounters for males (*4*).

	Weekly frequency of sexual encounters for males					
Age groups	0	1	2	3	4	5
15–24	0.167	0.167	0.229	0.229	0.104	0.104
25–29	0.109	0.463	0.1855	0.1855	0.0295	0.0275
30–39	0.201	0.473	0.134	0.134	0.029	0.029
40–49	0.254	0.51	0.0995	0.0995	0.0185	0.0185
50–59	0.456	0.383	0.075	0.075	0.0055	0.0055
60–69	0.551	0.354	0.0475	0.0475	0	0
≥70	0.784	0.15	0.029	0.029	0.004	0.004

Appendix Table 3. Age-dependent probability matrix of sexual encounters for females (*5*).

	Weekly frequency of sexual encounters for females					
Age groups	0	1	2	3	4	5
15–24	0.265	0.147	0.1765	0.1765	0.1175	0.1175
25–29	0.151	0.477	0.176	0.176	0.01	0.01
30–39	0.228	0.502	0.1095	0.1095	0.0255	0.0255
40–49	0.298	0.466	0.104	0.104	0.0135	0.0145
50–59	0.457	0.362	0.0845	0.0845	0.0055	0.0065
60–69	0.579	0.359	0.031	0.031	0	0
≥70	0.789	0.183	0.007	0.007	0.007	0.007

Appendix Table 4. Age-specific fertility rates per 10,000 women of reproductive age (*16*).

Country	Age 15–19	Age 20–24	Age 25–29	Age 30–34	Age 35–39	Age 40–44	Age 45–49
Belize	69.7	150.9	142	98.5	49.3	16.2	1.3
Bolivia	72.6	146.9	148.6	115	80.9	36.5	8
Brazil	68.4	107.6	90.6	55.8	29.2	10.2	1.9
Colombia	57.7	112.3	96.8	65	37.7	14.7	2.7
Costa Rica	59.1	101.1	87.2	70.3	40.5	10.7	1.3
Ecuador	77.3	139.3	124.6	90.9	55.1	24.4	5.7
El Salvador	66.8	108.1	97.6	70.5	37.2	12.6	1.7
French Guiana	82.6	156.2	182.5	151.3	88.7	33	2.6
Guatemala	84	173.2	159.4	124.2	80.1	32.8	6.4
Guyana	90.1	156.3	118.7	87.2	49.7	13.1	4.7
Honduras	68.4	134.8	113.7	87.3	56.2	28.2	5.3
Mexico	66	126.4	127.5	83	44	9.2	1.8
Nicaragua	92.8	122.5	108.7	76.1	42.8	16.1	4.6
Panama	78.5	149.1	132.2	87.9	38	9.1	0.9
Paraguay	60.2	129.8	130.3	102.9	65.3	26.1	5.1
Peru	68	110	113	104	73	25	3
Suriname	48.1	117	128.6	101.1	59	24.3	1.9
Venezuela	80.9	131.7	119	86	45.6	15.5	2.2

Appendix Table 5. Parameter values and their associated ranges used for simulations.

Parameter description	Baseline value (range)	Source
Human infection parameters		
Intrinsic incubation period	Mean: 5.7 d (lognormal);<br>shape = 1.72; scale = 0.21	(*6,7*)
Infectious period	Mean: 4.7 d (lognormal);<br>shape = 1.54; scale = 0.12	(*3,11*)
Risk of infection through sexual encounter	1%–5%	(*3*)
Fraction of infected cases experiencing asymptomatic infection	40%–80%	(*8,9*)
Mosquito lifespan and infection parameters		
Seasonal lifespan determined by a hazard function	Mean for high temperature season: 19.6 d<br>Mean for low temperature season: 11.2 d	(*3*)
Extrinsic incubation period	Mean: 10 d (lognormal);<br>shape = 2.28; scale = 0.21	(*12*)
Cost-effectiveness rates		
Disability weight for microcephaly (severe intellectual disability)	0.16 (1 case of microcephaly has 0.16 DALY)	(*13*)
Annual discount rate	3%	Assumed

Appendix Table 6. Direct life-time medical costs, and the per capita GDP for each country (*15*).

Country	Microcephaly	GBS	Physician visit for symptomatic cases	Per capita GDP (average of 2015–2016)
Belize	$103,586	$32,709	$61	$4,955
Bolivia	$80,974	$25,569	$57	$3,097
Brazil	$100,068	$31,599	$57	$8,694
Colombia	$78,990	$24,943	$68	$5,900
Costa Rica	$124,203	$39,220	$63	$11,563
Ecuador	$98,759	$31,185	$60	$6,084
El Salvador	$124,203	$39,220	$63	$3,719
French Guiana	$91,925	$29,027	$65	$18,036
Guatemala	$91,173	$28,790	$59	$4,032
Guyana	$98,974	$31,253	$57	$4,325
Honduras	$88,351	$27,899	$57	$2,358
Mexico	$93,867	$29,640	$67	$8,867
Nicaragua	$72,383	$22856	$56	$2,109
Panama	$107,620	$33,983	$63	$14,009
Paraguay	$81,542	$25,749	$58	$4,094
Peru	$88,850	$28,056	$61	$6,042
Suriname	$95,294	$30,091	$63	$7,298
Venezuela	$120,582	$38,076	$69	$7,766

Appendix Table 7. Mean ICER values with 95% confidence intervals corresponding to VCPI values under which vaccination program is at least 90% cost-effective in each country.*

	Very cost-effective			Cost-effective		
Country	VCPI	ICER	95% CI	VCPI	ICER	95% CI
Belize	$23	$3,516	$144–$4,575	$34	$12,092	$7,379–$15,050
Bolivia	$27	$1,827	$(872)–$2,669	$36	$7,038	$4,249–$9,745
Brazil	$21	$6,356	$1,596–$7,223	$38	$21,725	$14,938–$27,441
Colombia	$23	$4,184	$1,284–$5,349	$35	$14,086	$9,447–$16,736
Costa Rica	$16	$7,352	$1,280–$9,234	$29	$29,061	$15,459–$30,561
Ecuador	$32	$4,451	$1,343–$5,560	$48	$15,581	$10,338–$17,576
El Salvador	$26	$1,379	$(1,884)–$2,826	$34	$8,177	$3,408–$9,785
French Guiana	$47	$14,475	$10,016–$16,653	$96	$49,934	$36,523–$53,661
Guatemala	$32	$2,544	$148–$3,944	$45	$9,786	$6,556–$11,859
Guyana	$23	$2,270	$(285)–$3,717	$33	$10,034	$5,884–$12,262
Honduras	$23	$892	$(1,711)–$1,705	$29	$4,992	$1,623–$6,142
Mexico	$26	$6,362	$2,564–$7,445	$44	$21,652	$14,717–$24,875
Nicaragua	$18	$595	$(1,465)–$1,231	$24	$4,829	$2,395–$6,068
Panama	$43	$11,001	$7,016–$13,486	$82	$37,247	$29,096–$43,898
Paraguay	$23	$2,348	$(305)–$3,332	$32	$9,903	$5,028–$10,670
Peru	$22	$4,332	$1,087–$4,870	$35	$14,028	$9,262–$16,432
Suriname	$21	$4,434	$1,505–$6,235	$37	$18,705	$12,714–$22,331
Venezuela	$29	$4,697	$623–$6,590	$47	$19,170	$13,160–$23,579

*The per capita GDP and 3 times the per capita GDP were used as thresholds for very cost-effective and cost-effective analyses, respectively. The dollar values in parentheses indicate that the 95% CI extends to negative ICER values, which is considered cost-saving. Costs are in 2015 US dollars.

Appendix Table 8. Attack rates for additional simulation scenarios. The model was calibrated to each attack rate.

	Main scenario	Additional scenarios	
Country	Attack rate	Increase of 4% in baseline attack rate	Decrease of 4% in baseline attack rate
Belize	21%	25%	17%
Bolivia	10%	14%	6%
Brazil	18%	22%	14%
Colombia	12%	16%	8%
Costa Rica	2%	6%	1%
Ecuador	8%	12%	4%
El Salvador	16%	20%	12%
French Guiana	18%	22%	14%
Guatemala	14%	18%	10%
Guyana	15%	19%	11%
Honduras	14%	18%	10%
Mexico	5%	9%	1%
Nicaragua	17%	21%	13%
Panama	15%	19%	11%
Paraguay	17%	21%	13%
Peru	4%	8%	1%
Suriname	22%	26%	18%
Venezuela	19%	23%	15%

Appendix Table 9. Mean ICER values with 95% confidence intervals corresponding to VCPI values under which a vaccination program is at least 90% cost-effective in each country, with a 4% increase in attack rate.*

	Very cost-effective			Cost-effective		
Country	VCPI	ICER	95% CI	VCPI	ICER	95% CI
Belize	$24	$2,689	$(1,194)–$3,773	$34	$10,892	$6,136–$14,590
Bolivia	$30	$1,189	$(1,334)–$2,164	$40	$6,920	$4,089–$8,997
Brazil	$27	$6,589	$2,553–$7,720	$45	$21,841	$15,274–$26,353
Colombia	$26	$4,181	$1,458–$5,539	$38	$13,721	$9,371–$16,483
Costa Rica	$40	$9,072	$4,951–$12,098	$70	$30,013	$22,287–$33,976
Ecuador	$39	$3,618	$973–$5,276	$58	$15,088	$10,878–$18,087
El Salvador	$25	$1,098	$(2,753)–$2,733	$34	$7,545	$2,781–$10,230
French Guiana	$50	$14,914	$10,328–$18,865	$98	$49,466	$34,961–$53,192
Guatemala	$36	$2,197	$(200)–$3,521	$51	$10,076	$6,620–$11,936
Guyana	$27	$2,691	$(250)–$4,032	$37	$9,665	$5,907–$11,632
Honduras	$30	$1,078	$(1,445)–$1,723	$38	$5,439	$2,953–$6,806
Mexico	$43	$7,099	$4,304–$8,866	$70	$23,159	$18,270–$27,829
Nicaragua	$20	$1,067	$(757)–$1,789	$26	$5,069	$2,673–$6,063
Panama	$48	$10,427	$6,843–$13,151	$88	$34,894	$27,744–$42,041
Paraguay	$25	$2,662	$5–$3,705	$35	$9,702	$5,960–$11,045
Peru	$39	$4,398	$1,577–$5,465	$60	$15,565	$11,540–$17,911
Suriname	$21	$4,820	$798–$6,335	$35	$17,716	$11,223–$21,123
Venezuela	$34	$4,820	$1,944–$7,838	$51	$19,982	$11,823–$21,092

*The per capita GDP and 3 times the per capita GDP were used as thresholds for very cost-effective and cost-effective analysis, respectively. The dollar values in parentheses indicate that the 95% CI extends to negative ICER values, which is considered cost-saving. Estimates correspond to simulations calibrated to an increase of 4% in estimated attack rates for the 2015–2017 outbreaks. Costs are in 2015 US dollars.

Appendix Table 10. Mean ICER values with 95% confidence intervals corresponding to VCPI values under which vaccination program is at least 90% cost-effective in each country, with a 4% decrease in attack rate.*

	Very cost-effective			Cost-effective		
Country	VCPI	ICER	95% CI	VCPI	ICER	95% CI
Belize	$21	$2,344	$(812) –$3,581	$32	$12,128	$7,102–$14,544
Bolivia	$16	$909	$(1,459) –$2,077	$23	$7,207	$3,196–$8,751
Brazil	$21	$6,720	$2,642 –$8,089	$36	$20,704	$14,484–$24,808
Colombia	$16	$3,465	$266 –$4,008	$27	$14,476	$9,076–$17,082
Costa Rica	$9	$6,661	$(741) –$8,037	$18	$25,476	$12,133–$38,507
Ecuador	$19	$4,241	$688 –$5,265	$29	$13,608	$8,413–$16,282
El Salvador	$20	$1,183	$(1,846) –$2,852	$27	$8,404	$3,222–$9,843
French Guiana	$41	$15,037	$10,339 –$17,905	$84	$48,232	$37,689–$57,894
Guatemala	$25	$2,445	$(447) –$3,601	$35	$9,639	$5,399–$11,411
Guyana	$17	$2,130	$(1,099) –$3,429	$25	$10,149	$5,292–$13,610
Honduras	$16	$946	$(1,896) –$1,676	$21	$5,276	$1,658–$7,219
Mexico	$4	$3,054	$(5,722) –$2,798	$9	$19,550	$3,620–$23,927
Nicaragua	$14	$802	$(1,638) –$1,335	$19	$4,798	$2,246–$6,295
Panama	$29	$11,311	$5,967 –$13,785	$54	$34,281	$24,242–$41,282
Paraguay	$19	$2,627	$(29) –$3,344	$27	$8,492	$5,258–$10,724
Peru	$6	$2,594	$(2,114) –$2,779	$11	$13,487	$3,063–$17,903
Suriname	$18	$5,057	$1,164 –$6,269	$30	$16,836	$10,634–$20,560
Venezuela	$23	$4,915	$808 –$6,501	$39	$19,481	$12,735–$23,902

*The per capita GDP and 3 times the per capita GDP were used as thresholds for very cost-effective and cost-effective analysis, respectively. The dollar values in parentheses indicate that the 95% CI extends to negative ICER values, which is considered cost-saving. Estimates correspond to simulations calibrated to a decrease of 4% in estimated attack rates for the 2015–2017 outbreaks. Costs are in 2015 US dollars.

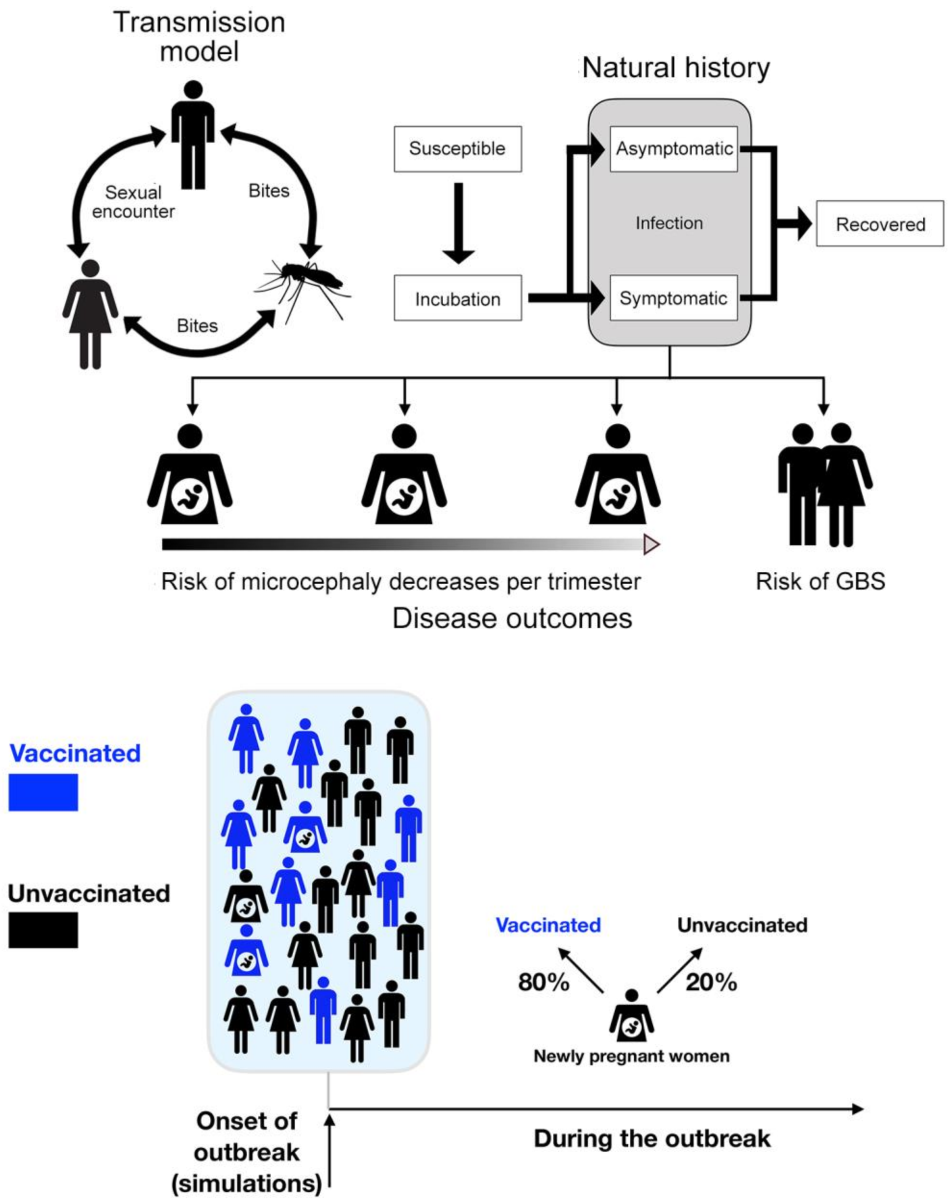

Appendix Figure 1. Schematic diagram for Zika virus model with vaccination.

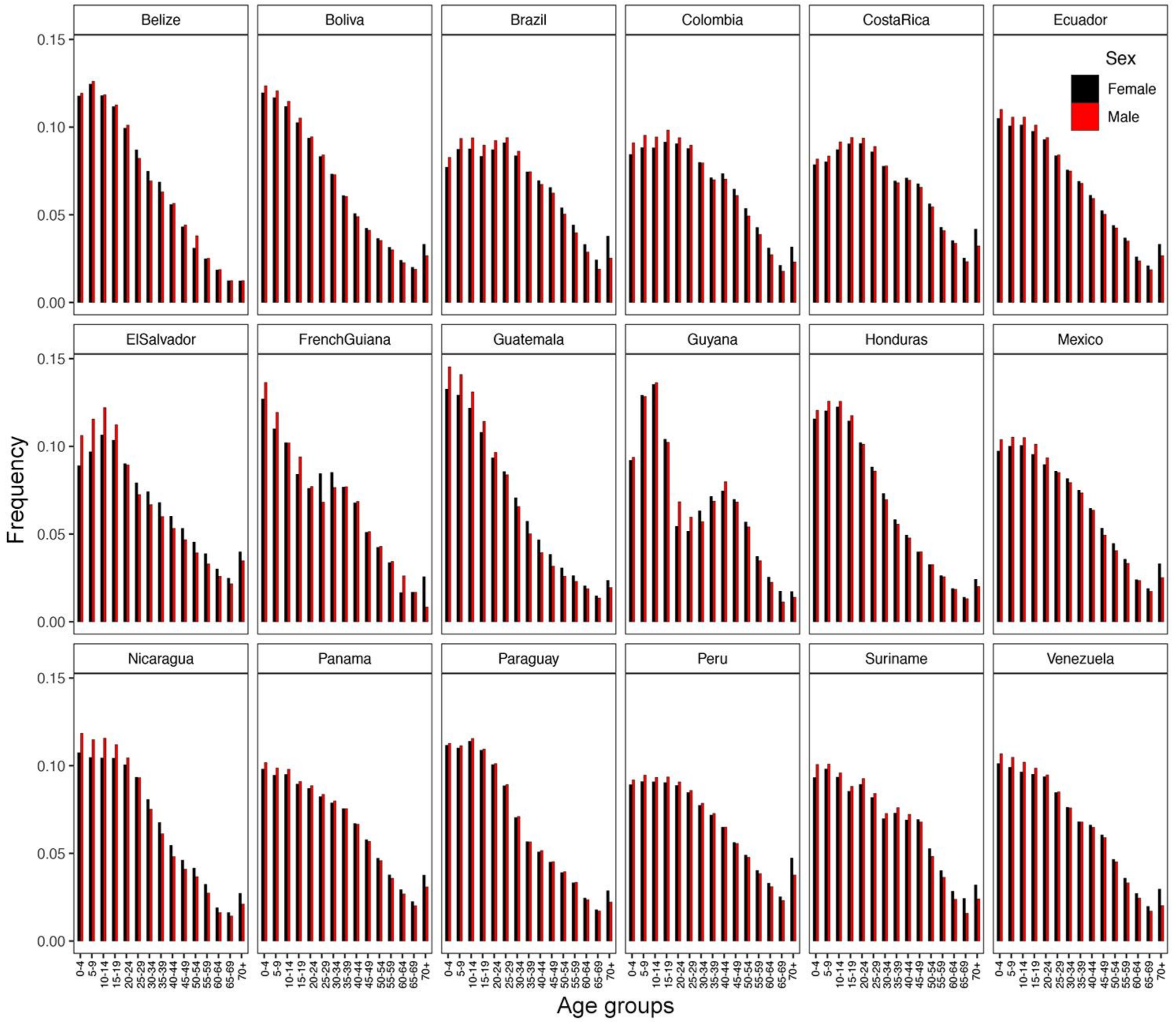

Appendix Figure 2. Age-sex distributions of human populations in various countries in Central and South America.

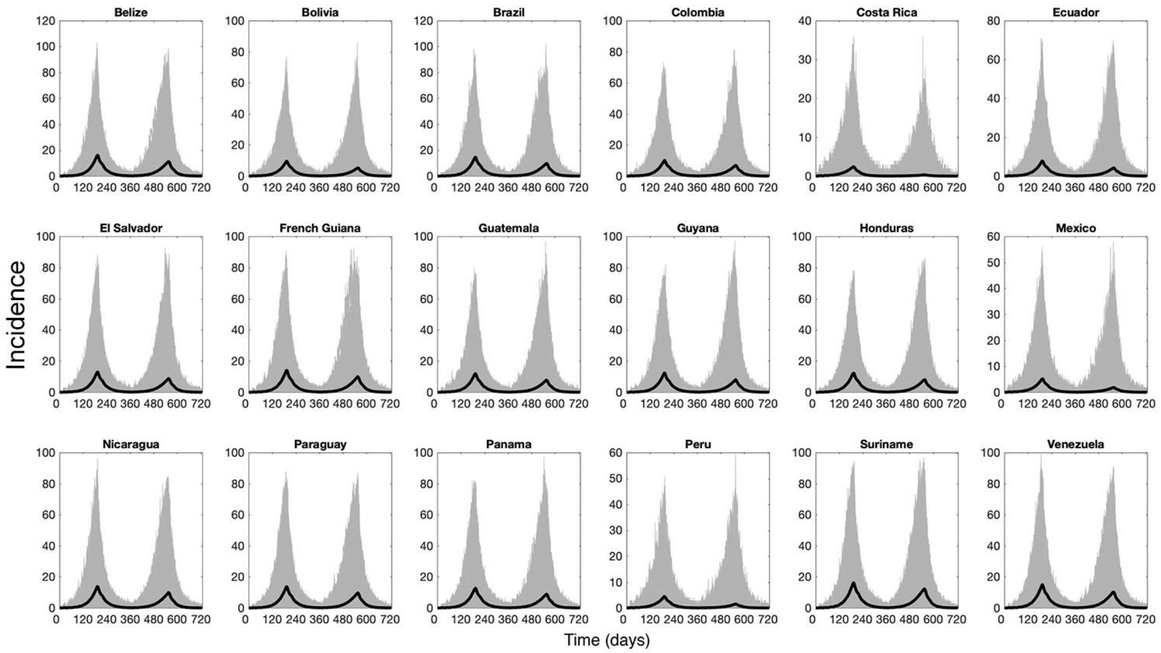

Appendix Figure 3. Incidence of Zika virus infection for each country with estimated attack rates for 2 years in the absence of vaccination (corresponding to the main scenario). The black curve shows the average of 2,000 realizations.

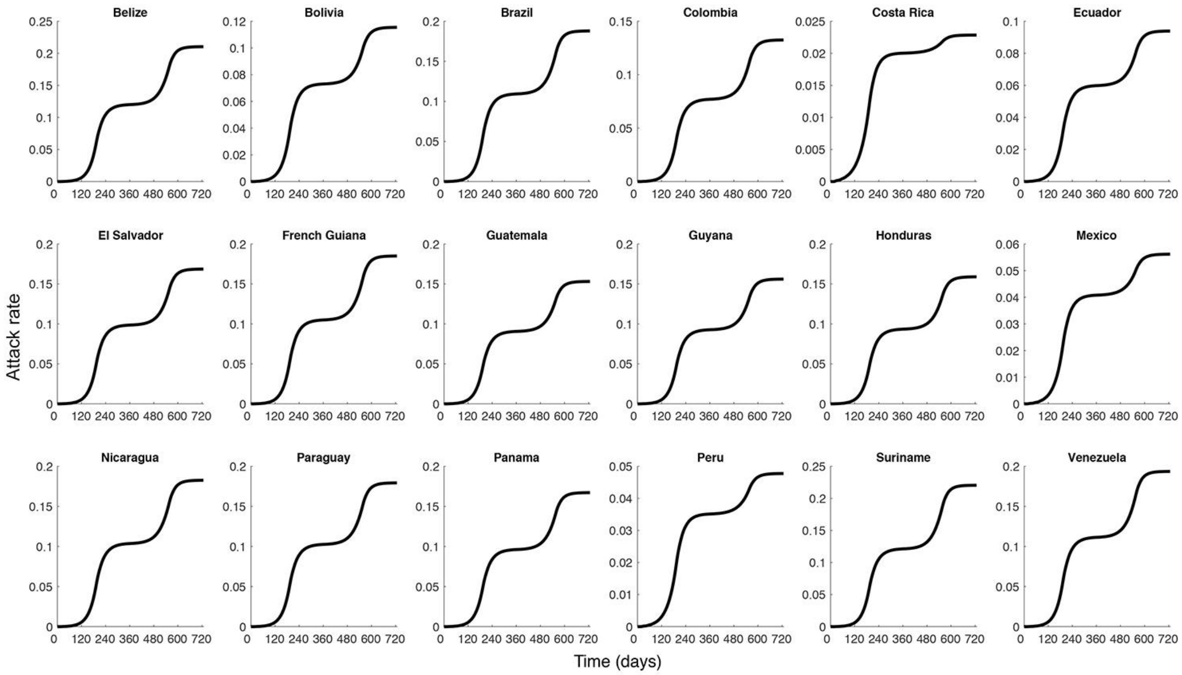

Appendix Figure 4. Attack rates (average of 2,000 realizations) of Zika virus outbreaks for 2 years in the absence of vaccination (corresponding to the main scenario).

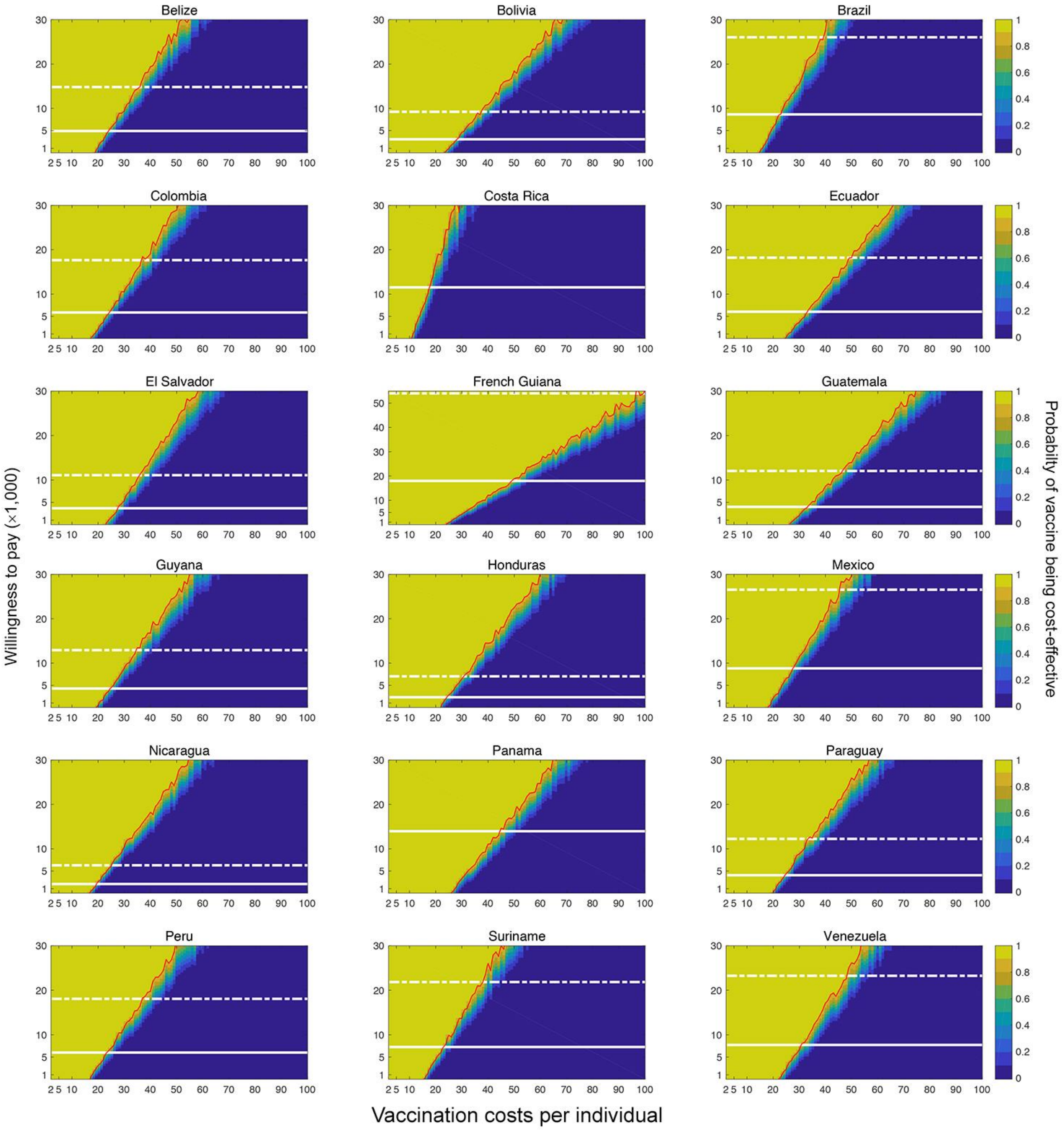

Appendix Figure 5. Probabilities of vaccine being cost-effective for a range of VCPI and willingness-to-pay. Solid white line represents the willingness-to-pay threshold corresponding to the average of per capita GDP of each country in 2015 and 2016. Dashed white line represents three times the average of per capita GDP of each country. The red curve represents the 90% probability of vaccine being cost-effective for a given VCPI (US dollars adjusted to 2015). GDP, gross domestic product; VCPI, vaccination costs per individual.

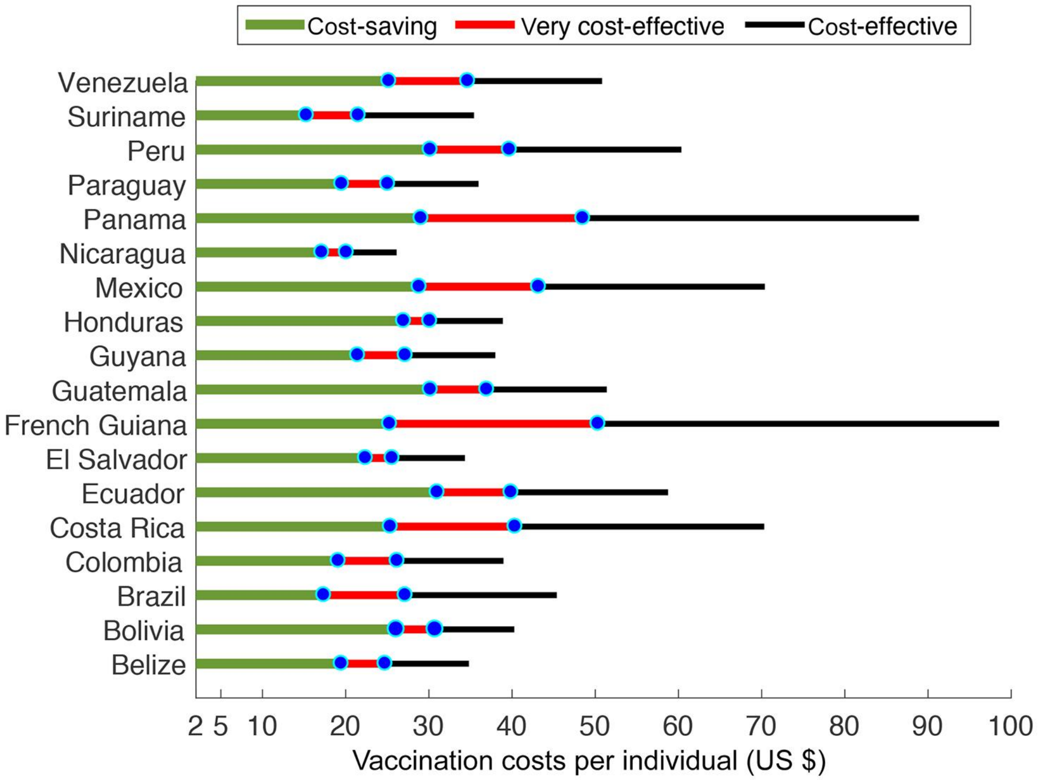

Appendix Figure 6. Vaccination costs per individual (in 2015 US dollars) for the scenarios of cost-saving (green), very cost-effective (red), and cost-effective (black). Estimates correspond to simulations calibrated to an increase of 4% in estimated attack rates for the 2015–2017 outbreaks.

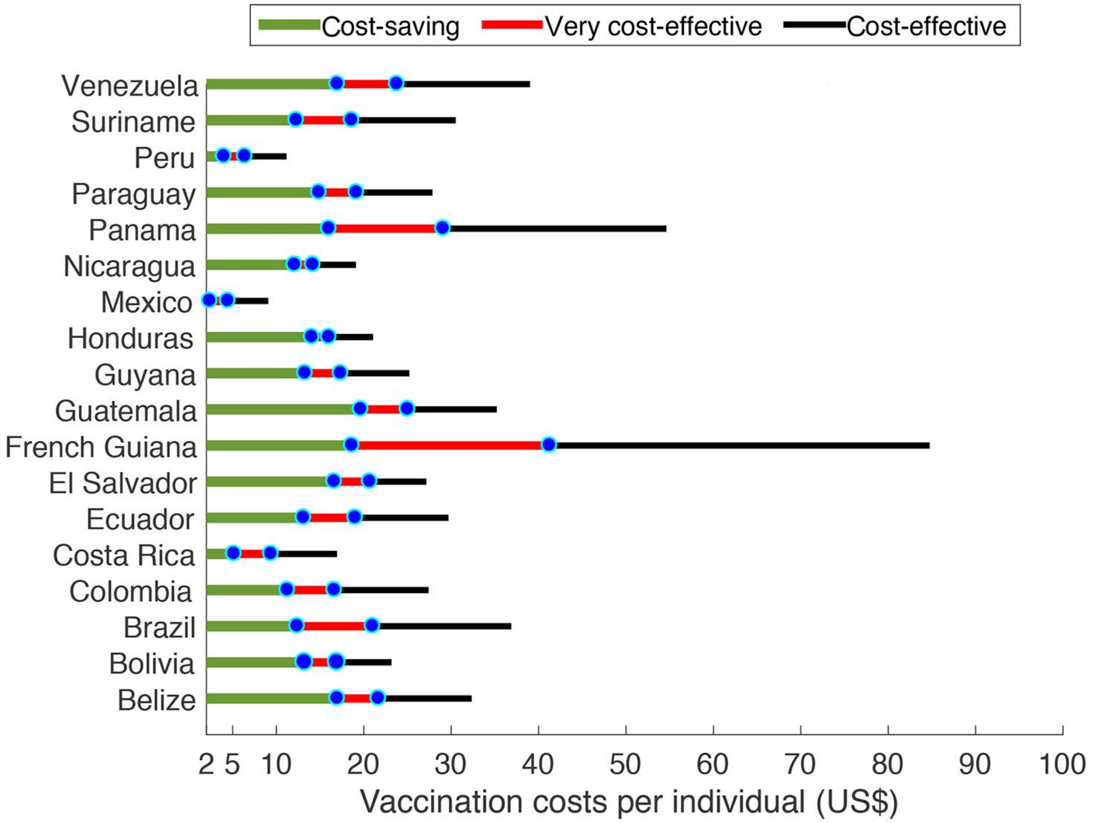

Appendix Figure 7. Vaccination costs per individual (in 2015 US dollars) for the scenarios of cost-saving (green), very cost-effective (red), and cost-effective (black). Estimates correspond to simulations calibrated to a 4% decrease in estimated attack rates for the 2015–2017 outbreaks.

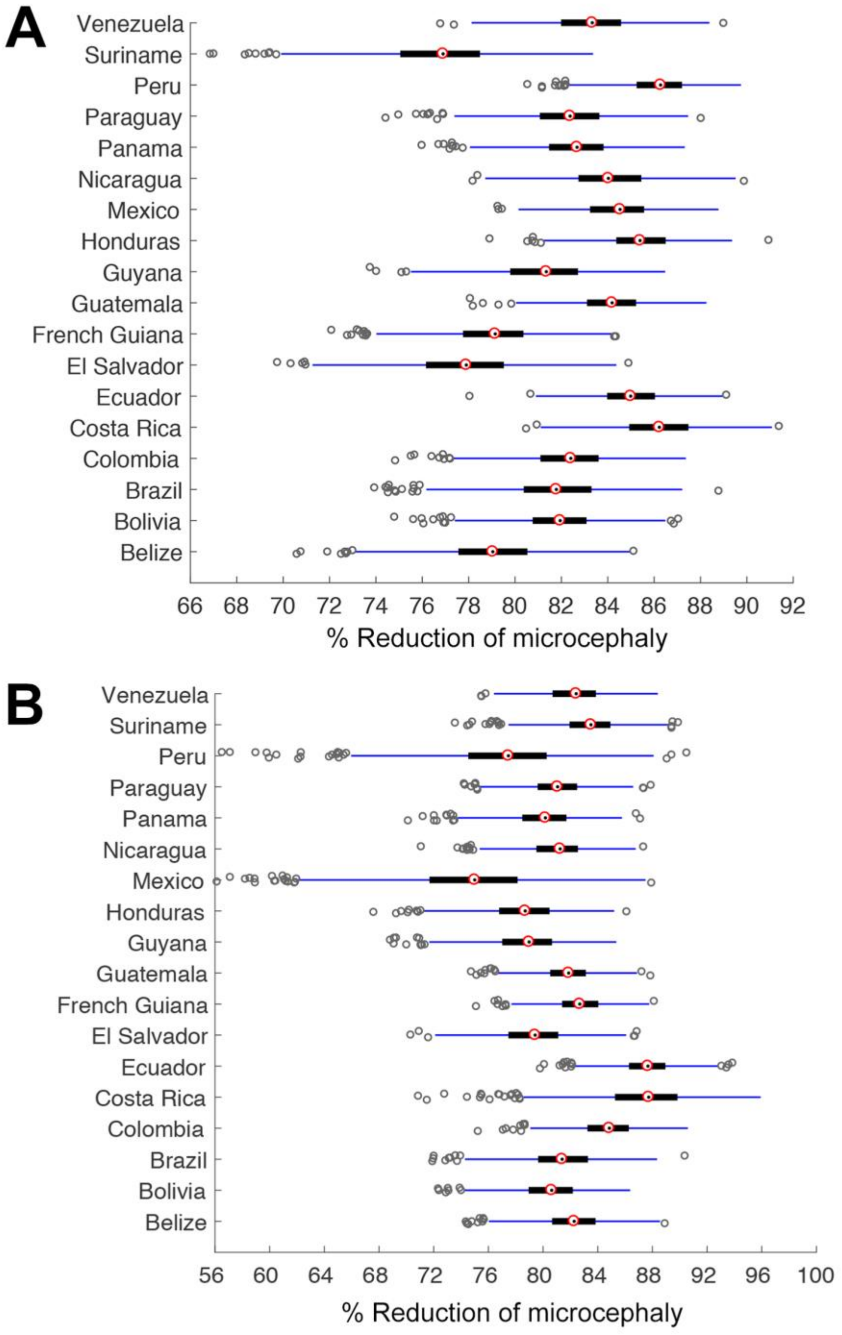

Appendix Figure 8. Box plots for the percentage reduction of microcephaly as a result of vaccination for A) an increase of 4% and B) a decrease of 4% in estimated attack rates for the 2015–2017 outbreaks. Medians are shown by the red circles.
